# The Effect of Specimen Width on the Deformation Behavior and Formability of cp-Ti Grade 4 Sheets During Uniaxial and Cyclic Bending Under Tension Loading

**DOI:** 10.3390/ma17235756

**Published:** 2024-11-25

**Authors:** Desmond Mensah, Nicholas Pitkin, Michael P. Miles, David T. Fullwood, Marko Knezevic, Brad Kinsey

**Affiliations:** 1 Department of Mechanical Engineering, University of New Hampshire, Durham, NH 03824, USA; desmond.mensah@unh.edu (D.M.); nicholas.pitkin@unh.edu (N.P.); marko.knezevic@unh.edu (M.K.); 2 Department of Manufacturing Engineering, Brigham Young University, Provo, UT 84602, USA; mmiles@byu.edu; 3 Department of Mechanical Engineering, Brigham Young University, Provo, UT 84602, USA; dfullwood@byu.edu

**Keywords:** uniaxial tension, cyclic bending under tension, digital image correlation, forming limit diagram

## Abstract

This study examines the specimen size-dependent deformation behavior of commercially pure titanium grade 4 (cp-Ti grade 4) sheets under tension, with strain paths between uniaxial tension (UT) and plane-strain tension and compares the results with cyclic bending under tension (CBT) data. Specimens of varying widths (11.7, 20, 60, 100, and 140 mm) were tested in both rolling (RD) and transverse (TD) directions. The research employed digital image correlation for full-field strain measurements, finite element simulations, and fracture surface thickness data. Contrary to traditional forming concepts, i.e., the forming limit diagram (FLD) has the lowest major strain at the plane-strain condition, and the fracture forming limit has decreased major strain with increasing (less negative) minor strain, wider specimens exhibited higher major strains at strain localization and fracture under UT. In contrast, CBT findings showed decreased formability with increasing width, i.e., closer to plane-strain deformation, as expected. Strain distribution analyses revealed a transition from nearly uniform deformation in narrow specimens to multiaxial strain states in wider specimens. Thickness measurements along the fracture surface revealed a steeper profile in UT compared to CBT, indicating more localized deformation and necking in UT. In comparison with AA6016-T4, the cp-Ti grade 4 showed greater thickness, suggesting lower susceptibility to localized thinning. Strong anisotropy was observed between the RD and TD, with TD specimens showing higher formability and steeper thickness gradients in UT. Strain fields, along with thickness reduction and adiabatic heating, are used to rationalize the observed width-sensitive deformation behavior of cp-Ti sheets. Notably, CBT improved overall formability compared to UT due to its ability to distribute strain more evenly and delay critical necking. The contrasting trends between simple UT and CBT emphasize the relationship between loading conditions, specimen geometry, and material behavior in determining formability. These findings highlight the ability of the CBT test to create known and desired deformation effects, i.e., lower major strain at failure with increasing specimen width, and more uniform deformation, i.e., consistent thinning across the specimen width, for cp-Ti. Given the observed effects of width in UT, the selection of the testing method is critical for cp-Ti to ensure that results reflect expected material behavior.

## 1. Introduction

Commercially pure titanium (cp-Ti) has become widely popular across various sectors, such as the aerospace, chemical processing, and medical industries, due to its strength, resistance to corrosion, and biocompatibility [1,2,3]. As the demand for complex-shaped titanium components increases, understanding the material’s formability and deformation behavior becomes crucial for optimizing manufacturing processes and accurately predicting forming limits, whether in terms of necking or fracture.

**Figure 1:**
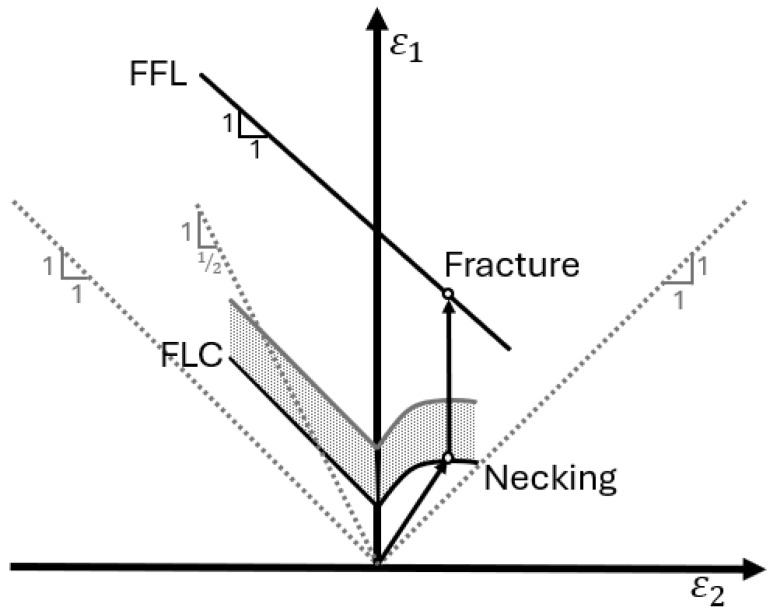
Schematic representation of the principal strain space showing the forming limit curve (FLC), the fracture forming limit (FFL), and an idealized strain path with arrows obtained in a sheet metal formability test utilized for determining these limit curves [6].

The concept of forming limit diagrams (FLDs) for plastic instability was first introduced by Keeler and Goodwin in the 1960s [4,5]. These diagrams have since become a cornerstone in sheet metal forming analysis, providing a graphical representation of the maximum allowable strains before the onset of localized necking. The boundary between safe deformation and failure in these diagrams is depicted by the forming limit curve (FLC). Similar to the onset of necking, the fracture forming limit (FFL) of the material is also critical to understand during forming process design. See Figure 1 for a generic depiction of FLC and FFL curves [6]. Traditionally, the characterization of sheet metal formability has relied heavily on FLDs and FFLs, which provide valuable insights into the maximum strains a material can withstand under various strain paths. The standard method for determining FLCs and FFLs involves testing sheet specimens of different shapes and widths to achieve various strain states, from uniaxial tension (UT) to balanced biaxial stretching [7], with the test simply progressing from the onset of localized necking (i.e., FLC) to fracture (i.e., FFL). While the shape of an FLC has an approximate “V” shape with the apex near the plane strain condition, the FFL typically has a decreasing slope, with increasing minor strain from uniaxial to biaxial stretching [6,8,9]. Testing methods assume that the FLC and FFL are intrinsic material properties independent of the loading path and specimen geometry [10].

However, studies have highlighted the limitations of conventional FLCs, particularly their sensitivity to strain path changes and specimen geometry [11,12]. Research has shown that the FLC can be influenced by factors such as sheet thickness, grain size, and specimen width [13,14,15,16,17]. (While not also investigated, FFLs would presumably show similar dependencies). The effect of specimen width on formability is particularly significant, as it directly impacts the stress state and strain distribution during deformation. The advent of advanced measurement techniques, such as digital image correlation (DIC), has allowed for a more detailed analysis of strain fields during deformation. These techniques have revealed that the assumption of a uniform strain state across the width of a specimen may not always hold, particularly for wider specimens [18,19].

The importance of sheet width in formability studies was recognized as early as the 1970s, with researchers noting that wider specimens tended to exhibit higher limit strains on the positive minor strain side of the FLD [20]. This observation led to the development of various specimen geometries for FLC determination, including the Nakajima and Marciniak tests, each attempting to capture a range of strain states by varying the specimen width [21,22]. More recent investigations have focused on the size-dependent nature of plastic deformation in sheet metals, which have shown that as the specimen width increases, there is a transition from a predominantly uniaxial stress state to a more complex, multiaxial stress state near the center of the specimen [23,24,25,26,27,28]. This transition can significantly affect the strain distribution and localization behavior, influencing the material’s forming limits.

The relationship between sheet thickness reduction and specimen width during loading is also an important aspect of formability analyses. As specimen width increases, the deformation mode transitions from UT towards plane strain conditions, significantly affecting the thickness reduction behavior. Tardif and Kyriakides observed that wider specimens tend to exhibit more uniform thickness reduction across their central region, while narrower specimens show more localized thinning [24]. This phenomenon is attributed to the constraint effect imposed by the increased width, which inhibits lateral contraction. Consequently, as noted by [29,30], the forming limit strains in wider specimens are often higher than those in narrower ones, partly due to this more distributed thickness reduction. Furthermore, Xu et al. [17] demonstrated that the rate of thickness reduction varies non-linearly with specimen width, with a critical width beyond which the deformation at the center of the specimen approaches plane strain conditions. In this state, the strain in the width direction (minor strain) becomes negligible, and deformation primarily occurs through thickness reduction and elongation in the loading direction. This width-dependent thickness reduction behavior has significant implications for forming limit predictions, as it influences the strain paths and stress states experienced by the material during deformation.

Previous research on cyclic bending under tension of cp-Ti sheets [31] revealed insights into the width-dependent behavior of titanium under combined bending and tensile loading conditions. In that study, it was observed that the elongation-to-fracture in CBT decreased significantly as specimen width increased. This trend was attributed to a shift in the strain path from UT in narrow specimens towards plane-strain conditions in wider specimens. Notably, while CBT improved formability across all widths compared to simple tension, the magnitude of this improvement diminished with increasing specimen width. DIC and finite element simulations of the CBT process indicated that wider specimens experienced reduced width strains and increased thinning strains, mirroring trends observed in forming limit curves. These findings highlighted the effects of cyclic deformation and specimen geometry on cp-Ti. Building upon these CBT results, this present study on simple tension across various specimen widths aims to isolate and quantify the geometric effects on deformation behavior without the added complexity of cyclic loading and then compares the UT and CBT findings.

In this study, experimental work (with DIC techniques for high-resolution strain analysis) and numerical simulations (using a commercial finite element (FE) solver, Abaqus/Implicit 2023) are employed to explain the relationship between specimen geometry and material response under monotonic UT loading, as well as CBT processing and its influence on formability. The study examines how specimen width affects key properties, i.e., tensile strength and engineering strain at failure, as well as how width influences strain paths and localization patterns. For FLCs, the CBT tests on cp-Ti and the UT tests on AA6016 both demonstrate the expected decreasing strain trend with increasing width for specimen sizes of 11.7, 20, and 60 mm. But UT of cp-Ti does not, nor UT for 100 and 140 mm specimen widths, for either material. In contrast, for FFL, the CBT tests on cp-Ti show the expected decreasing strain trend with increasing widths for all width specimens. However, for both cp-Ti and AA6016, UT testing shows increasing strain at fracture with increasing width, which is contrary to known material behavior. This decreasing engineering strain trend with increasing width at fracture demonstrates a benefit to the CBT testing method, in particular for cp-Ti, including achieving more uniform deformation, i.e., consistent thinning, across the specimen width.

The subsequent sections present the detailed experimental methodology (including DIC strain analysis), numerical simulation procedures using Abaqus, analyses of results, and discussion of the implications for forming limit predictions, both the onset of necking and fracture. The findings of this study, including the ability of the CBT test to produce lower major strain at failure with increasing specimen width and consistent thinning across the specimen width for cp-Ti, have significant implications for the sheet metal forming industry, particularly in the context of titanium processing by demonstrating the importance of considering specimen geometry and testing method in formability assessments.

## 2. Materials and Methods

### 2.1. Material

**Figure 2:**
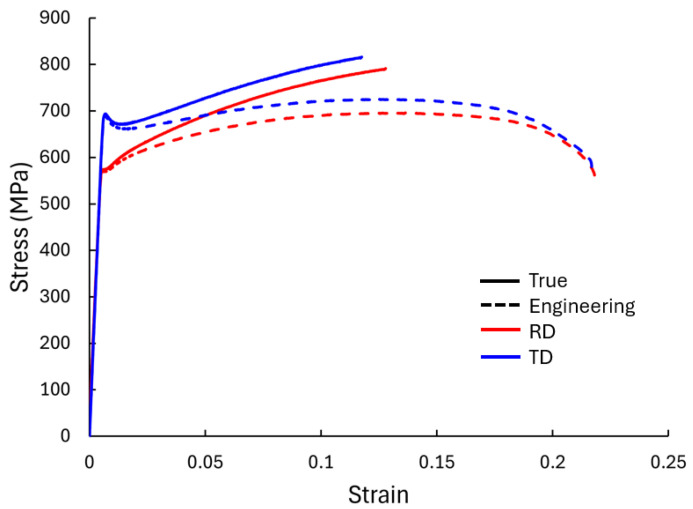
Stress–strain curves of as-received 1 mm sheets of cp-Ti measured along the rolling (RD) and transverse (TD) directions during standard uniaxial tension under 0.001/s strain rate at room temperature.

The study was conducted on 1 mm-thick cp-Ti grade 4 sheets (supplied by Boeing under specification: AMS-T-9046 CP-1 (grade 4) in an annealed condition), which was the focus of this work, as well as 1 mm-thick AA6016-T4 sheets, which are presented for comparison purposes as the microstructural deformation mechanisms between these two materials are considerably different. The chemical composition of the cp-Ti is provided in Table 1. The strength and ductility properties of the as-received material were previously characterized by Oishi et al., 2024 [31], in the rolling direction (RD) and transverse direction (TD). The mechanical properties of the cp-Ti material from a modified ASTM E8 [32] UT specimen geometry, i.e., 75 mm gauge length, are summarized in Table 2 and shown in Figure 2. These data illustrate the anisotropic behavior of the cp-Ti grade 4 sheet, with notable differences in mechanical properties between the two tested directions. The TD shows the highest yield and ultimate tensile strength. The initial microstructure of the cp-Ti sheets has been characterized by EBSD analysis and shown in Appendix A [33,34]. The chemical composition and basic properties of AA6016-T4 can be found in [35].

### 2.2. Specimen Preparation

**Figure 3:**
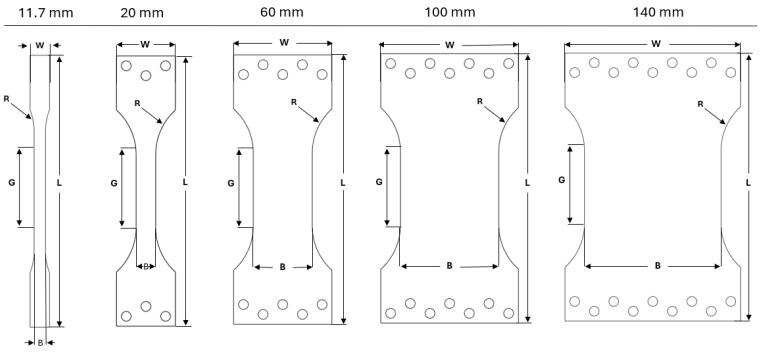
Geometries of the uniaxial tension specimens for all widths: Table 3 contains the dimensions for each sample and explanation of the alphabetical labels.

Cp-Ti and AA6016 sheets were machined into testing specimens with five different widths (11.7, 20, 60, 100, and 140 mm) in both the RD and TD with an abrasive water jet cutting process. The edges of the specimens were sanded smoothly to prevent premature fracture. Figure 3 illustrates the various specimen geometries, with specific dimensions listed in Table 3. The range of specimen widths was selected to investigate the effect of size on the deformation behavior and formability under UT and CBT testing. The narrowest specimen (11.7 mm) represents a standard UT test geometry [32] with a modified gauge length, while the widest specimen (140 mm) approaches a plane-strain condition in the center. Tests were repeated at least twice, but in some cases only once due to the lack of material for wide specimen widths.

### 2.3. Experimental Setup

**Figure 4:**
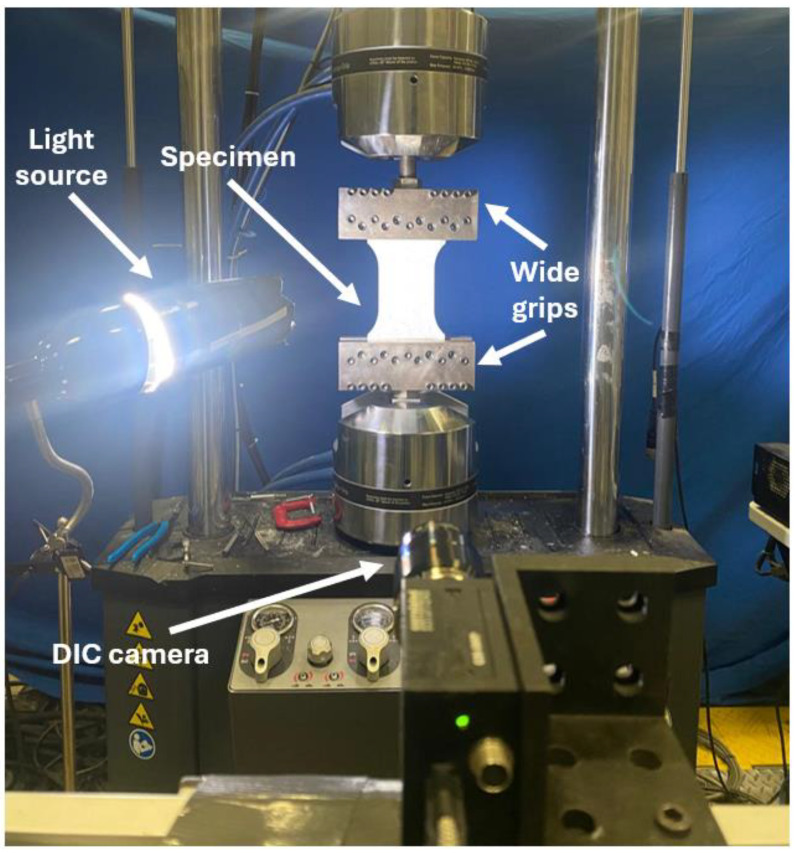
Experimental setup in MTS with a DIC camera and light source for strain measurements.

The UT tests for specimens of various geometries were conducted using a 250 kN MTS Landmark 370 servo-hydraulic universal testing machine (MTS Systems Corporation, 14000 Technology Drive, Eden Prairie, MN 55344, USA), as shown in Figure 4. The crosshead speed was set at 0.075 mm/s for all tests, which produced an initial strain rate of ~0.001/s. Custom fabricated wide grips enabled the testing of wide sheet specimens, in addition to standard UT grips [32] for 11.7 mm specimens. To obtain full strain field measurements, a 2D DIC system was employed with a random black speckle pattern applied on a white background to the sample surfaces. The DIC system consisted of a 9 MP Grasshopper3 89S6M camera (FLIR Systems, Inc., 27700 SW Parkway Ave., Wilsonville, OR 97070, USA) and a light source for uniform illumination of the specimen surface. To ensure an accurate correlation between load and strain data, the DIC system was synchronized with the MTS load cell throughout the test. Post-processing of the captured images was performed using VIC-2D version 6 software from Correlated Solutions^®^ (Correlated Solutions, 121 Dutchman Blvd., Irmo, SC 29063, USA) with a filter size of 15, a subset of 29 pixels, and a step size of 7 pixels for strain and distribution analyses.

### 2.4. FE Simulation Setup

Finite element (FE) simulations were conducted using Abaqus 2023 software to complement the experimental investigations and provide additional insights into the stress and strain distributions across different specimen widths. The sheets were modeled using C3D8R elements (8-node linear, reduced integration brick elements), with an isotropic plasticity material model based on the experimental UT data from both the RD and TD. Four elements were used through the thickness for all specimen widths to ensure adequate resolution of the deformation in this direction. The in-plane mesh density was varied according to the specimen width, with 8, 17, 29, 43, and 57 elements used across the width for the 11.7, 20, 60, 100, and 140 mm wide specimens, respectively. Taking advantage of the symmetry in the specimen geometry and loading conditions, a half-symmetry model was employed along the specimen length, as shown in Appendix B. The loading conditions in the simulations were designed to replicate the experimental setup, with one of the grip regions fixed and displacement-controlled boundary conditions applied to the other grip region. The simulation was run until a significant localization of plastic strain occurred, which emulates the onset of necking.

## 3. Results

### 3.1. Effect of Specimen Width on Stress–Strain Behavior

**Figure 5:**
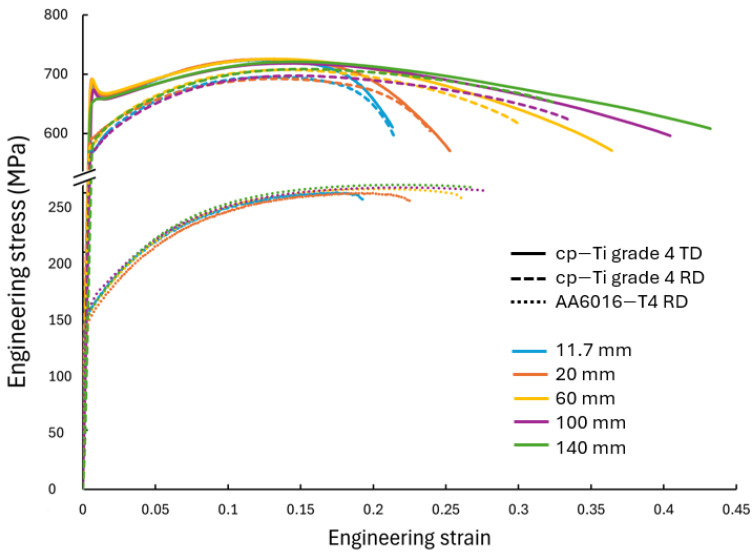
“Apparent” engineering stress–strain curves of uniaxial tension of cp-Ti grade 4 RD, cp-Ti grade TD, and AA6016-T4 RD at a strain rate of ~0.001/s.

Figure 5 presents the “apparent” engineering stress–strain curves for cp-Ti specimens of varying widths in the RD and TD, as well as for AA6016 in the RD, where the strain data were obtained using a virtual extensometer in the VIC-2D version 6 software, measuring over the gauge length in the center of the specimen. Due to the non-standard specimen geometries used in this study, particularly the varying widths, these curves represent the global response of the specimens but may not reflect the local stress–strain state at every point, especially for wider specimens where stress and strain distributions are complex. We use the term “apparent” to acknowledge that while these curves were calculated using standard engineering stress and strain definitions, they represent average measures that may not fully capture the complex stress and strain distributions, particularly in wider specimens.

**Figure 6:**
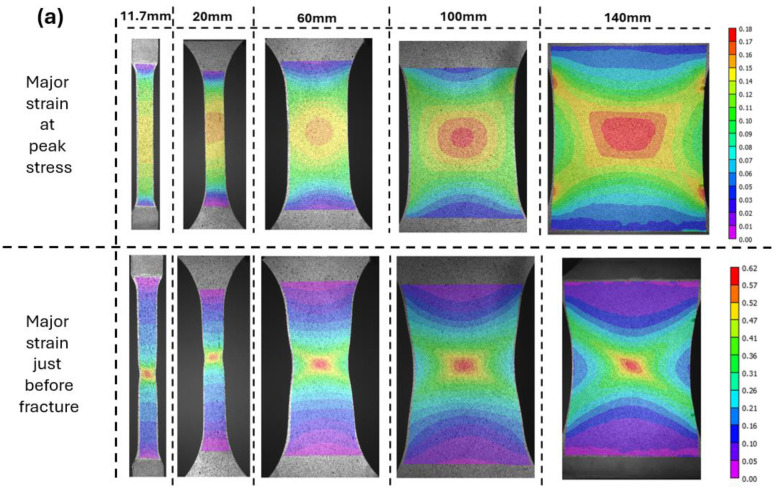

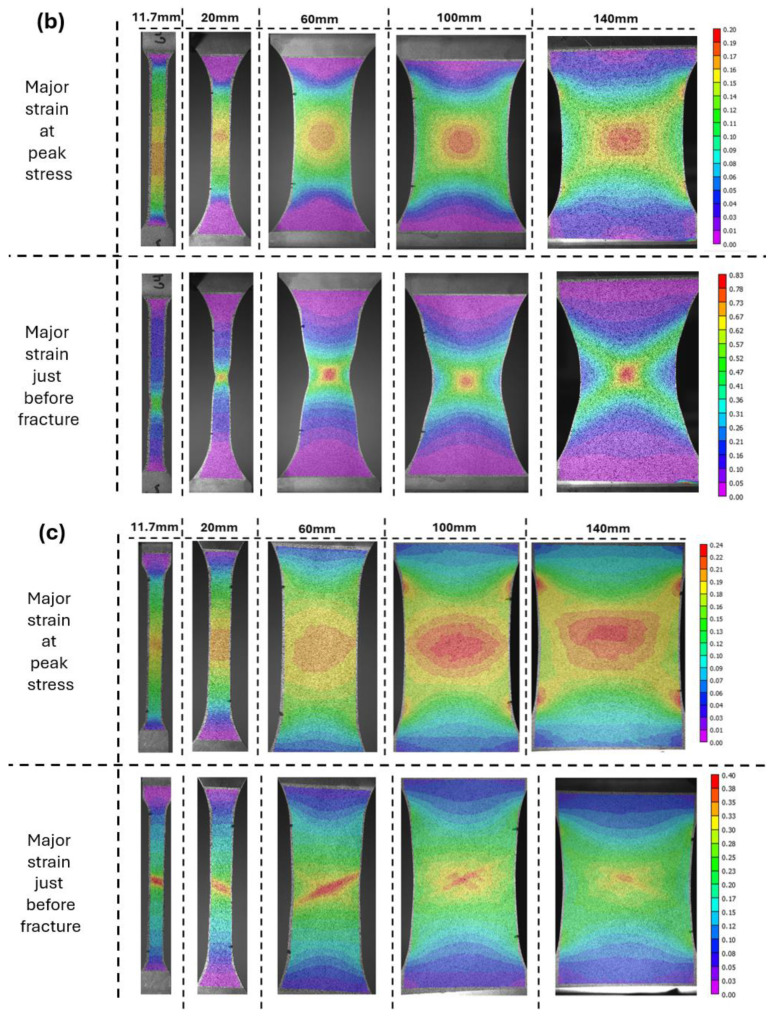

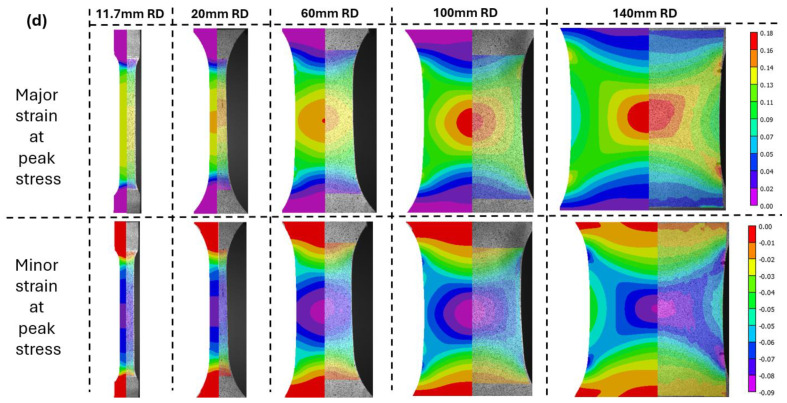
DIC strain contour plots at peak stress and just before fracture across different widths for (**a**) cp-Ti grade 4 RD, (**b**) cp-Ti grade 4 TD, and (**c**) AA6016-T4 RD, as well as (**d**) a comparison between cp-Ti grade 4 RD experiments and simulations at peak stress.

There was a clear trend observed, i.e., as the specimen width increased, the engineering strain at fracture increased. The yield strength and ultimate tensile strength were relatively consistent across all specimen widths in both directions of cp-Ti, indicating that the peak stress was less sensitive to the width. However, the post-necking behavior showed marked differences. Wider specimens demonstrated a more gradual decrease in stress after the peak stress, suggesting a more stable necking process and more gradual strain localization. The cp-Ti TD samples showed slightly higher yield and ultimate tensile strengths compared to the cp-Ti RD samples, consistent with the anisotropic behavior typically observed during UT performed on ASTM E8 standard specimens (Figure 2) [31]. The width effect on engineering strain at fracture can be attributed to several factors. Figure 6 shows the strain distribution patterns, which varied significantly with specimen width. In narrower specimens (11.7 mm and 20 mm), the strain distribution was nearly uniform across the width (although slight strain concentrations along the length of the specimen are evident for the cp-Ti TD and AA6016 specimens, even for the 11.7 mm width). As specimen width increased, a more complex strain distribution emerged, with the widest specimens (100 mm and 140 mm) showing a distinct gradient from the center to the edges. The strain localization, which leads to fracture, occurred over a larger area in wider specimens, contributing to their higher overall strain capacity and integrity before fracture. Similarly, as specimen width increased, there was a gradual transition from a uniaxial stress state to a more complex stress state across the width of the specimen. The ability of the wider sheets to accumulate the strain/stress gradient is beneficial. This gradient transition from the middle of the sheet specimen to the surrounding material delayed the onset of necking and increased the overall engineering strain at failure. These effects will be discussed further in later sections.

### 3.2. Effect of Specimen Width on Strain Paths to Fracture

**Figure 7:**
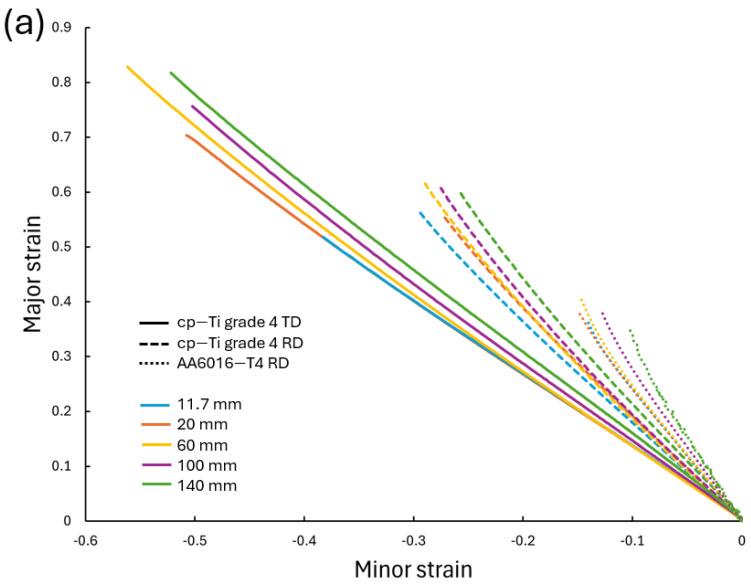

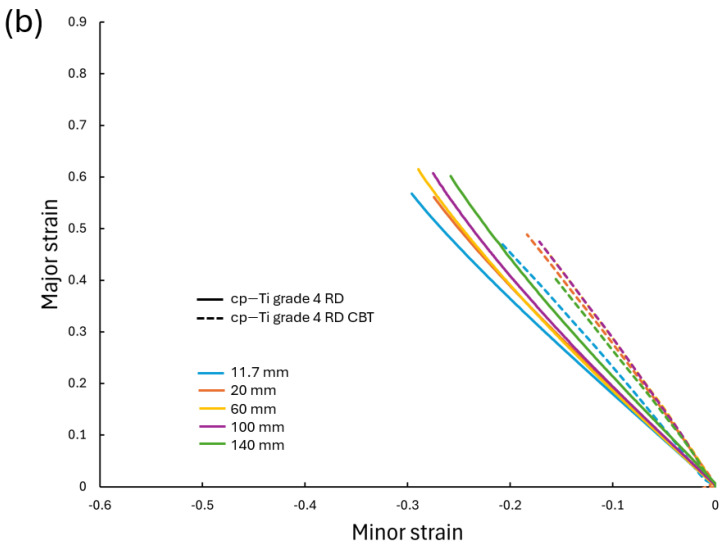
Strain paths to failure for all specimen widths of (**a**) cp-Ti grade 4 RD, cp-Ti grade TD, and AA6016-T4 RD under UT, and (**b**) cp-Ti grade 4 RD under UT and CBT loading.

The strain paths to failure are presented in Figure 7a for cp-Ti in the RD and TD, as well as for AA6016 in the RD for comparison. As the width increased from 11.7 mm to 140 mm, the strain paths became steeper, indicating a shift towards plane strain conditions. The TD samples demonstrated higher major strains at fracture compared to the RD samples, consistent with the higher engineering strain at failure observed in the stress–strain curves. The AA6016 shows a similar trend in strain path evolution with width but with lower overall strain values and paths closer to plane strain.

Figure 7b shows the strain path for the cp-Ti RD under both UT and CBT loading. Similar trends were observed, except for the 140 mm wide CBT test, which was due to difficulties with this size specimen in the experimental setup [31]. While CBT testing produced lower local strain values at fracture, more uniform deformation, with respect to the width and along the gauge length, was achieved, as will be shown later by thickness measurements. Note that the goal in both UT and CBT tests was to achieve a condition closer to plane strain with wider specimens, but the heterogeneous deformation shown in Figure 6 and [31] prevented a true plane-strain condition from occurring.

**Figure 8:**
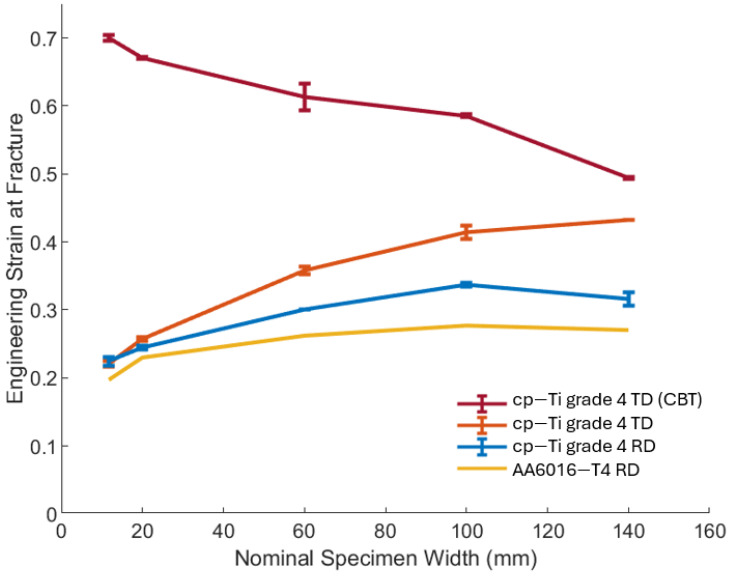
Effect of specimen width on engineering strain at fracture for cp-Ti grade 4 in UT the RD and TD, as well as the CBT RD, and the AA6016-T4 RD.

The relationship between nominal specimen width and engineering strain at fracture for cp-Ti in the RD and TD, as well as AA6016 in the RD, is shown in Figure 8. For all materials and directions, there was a general trend of increasing strain at fracture with increasing specimen width. Also plotted in Figure 8 is the engineering strain at fracture for CBT experiments of the cp-Ti RD [31], which showed decreasing engineering strain with increasing width. This was consistent with the FFL, where a decreasing strain with increasing minor strain was expected. Also, note that despite this decreasing trend, CBT strains remained higher than those in UT across all widths since the CBT process enhances material formability. The trend of increasing strain at fracture with increasing specimen width for both cp-Ti and AA6016 UT in the RD was most pronounced up to the 100 mm width, after which it appeared to plateau or slightly increase in the case of the cp-Ti TD. Higher engineering strains at fracture were exhibited in the TD compared to the RD across all widths, with the difference becoming more pronounced for wider specimens. Note that the difference in the CBT strain values at fracture between Figure 7b (i.e., the end of the strain path curves) and Figure 8 was the DIC method used to obtain the data, i.e., a digital extensometer along the gauge length and a DIC strain point at the fracture location, respectively.

**Figure 9:**
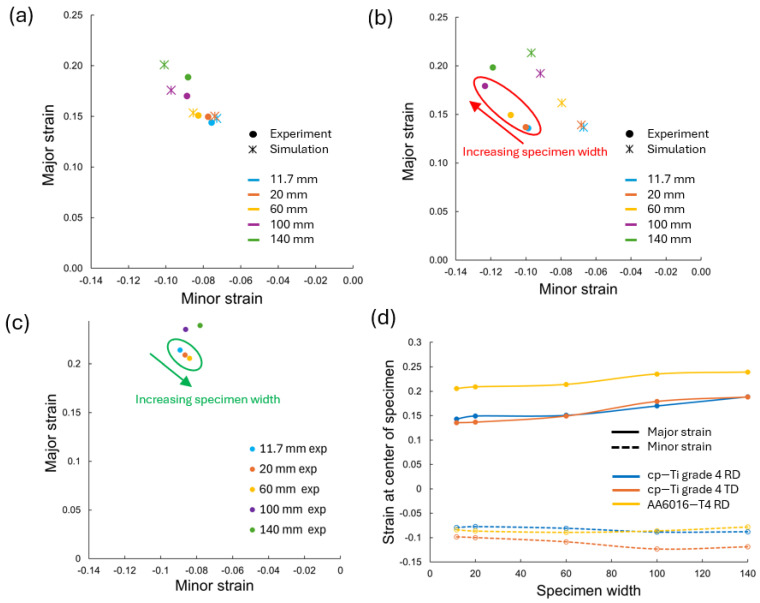
Limit strains at peak engineering stress values, i.e., necking, for all specimen widths of (**a**) cp-Ti grade 4 RD, (**b**) cp-Ti grade TD, and (**c**) AA6016-T4 RD; (**d**) major and minor limit strains at the center of specimens for all materials and direction.

Figure 9 presents the limit strain states at the peak stresses, i.e., necking, for cp-Ti in the RD and TD, as well as the AA6016 RD, comparing experimental results with finite element simulations for the cp-Ti cases. Both the RD and TD for cp-Ti showed an increasing trend in major limit strain with specimen width. (See the red oval, arrow, and text in Figure 9b). The increase was more pronounced for widths above 60 mm. As previously noted, the TD consistently exhibited higher major limit strains than the RD. Minor limit strains showed a decreasing trend with increasing width for both the RD and TD, although not all data followed this behavior due to the variability of strains at the peak stresses, e.g., 100 mm and 140 mm data for cp-Ti. The rate of change in major and minor limit strain with width was more evident in the cp-Ti TD compared to the cp-Ti RD, as there was a greater spread in the data points. FEA predictions for cp-Ti showed the expected pattern clearly. From Figure 9d, which compares the three cases in the same plot, AA6016 had the highest strain value at the peak stress. However, as shown in Figure 8, the strain at fracture was lowest for AA6016. This further highlights the greater post-localization deformation achieved in cp-Ti compared to AA6016.

Note that this increasing major limit strain and decreasing minor limit strain with increasing specimen width for cp-Ti is opposite to the trend predicted in FLD or FFL tests in past literature for both experimental and analytical modeling, e.g., with the M-K analysis. For AA6016, the expected FLD trend was obtained for 11.7, 20, and 60 mm specimen widths with decreasing major strain and increasing (less negative) minor strain, as expected. (See the green oval, arrow, and text in Figure 9c). However, the significant heterogeneous deformation pattern for 100- and 140 mm-width specimens prevented these trends from continuing to hold as the strain path shifted closer to a plane-strain condition. This reinforces the importance of considering specimen geometry in formability assessments.

The strain ratio, which for a standard UT specimen geometry [32] is referred to as the *r* (Lankford) coefficient (but is not the appropriate terminology here due to the varying UT geometries), were computed using:

(1) $$r=\frac{\varepsilon_{2}^{p}}{\varepsilon_{3}^{p}}$$

where $\varepsilon_{3}^{p}$ is the logarithmic thickness strain computed from the major logarithmic strain $\varepsilon_{1}^{p}$ (along the loading direction) and $\varepsilon_{2}^{p}$, which is the minor logarithmic strain (along the transverse direction) considering volume conservation, i.e., $\varepsilon_{3}^{p}=-\varepsilon_{1}^{p}-\varepsilon_{2}^{p}$. Cp-Ti showed pronounced anisotropy, with notably higher strain ratios in the TD compared to the RD (Table 4). The TD exhibited a general trend of decreasing strain ratios as specimen width increases, particularly for wider samples, i.e., >60 mm. In contrast, the RD displayed a non-uniform pattern, with strain ratios fluctuating across different widths and reaching a peak at intermediate widths, although still decreasing for widths >60 mm. AA6016, on the other hand, demonstrated consistently lower strain ratios than cp-Ti, with a clear decline as the specimen width increased. These patterns indicated a shift in deformation modes with changing specimen geometry. The stark contrasts between cp-Ti and AA6016, as well as between the RD and TD of cp-Ti, emphasize that these width-size effects are material-specific.

### 3.3. Fracture Surface Thickness

**Figure 10:**
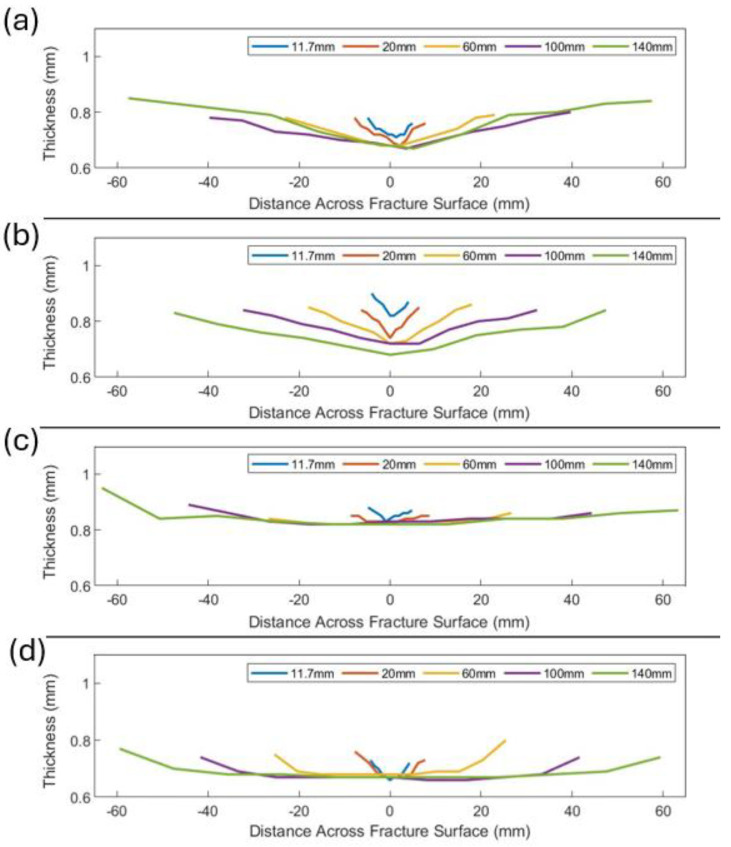
Fracture surface thickness variation across different widths for (**a**) cp-Ti grade 4 RD, (**b**) cp-Ti grade 4 TD, (**c**) AA6016-T4 RD, and (**d**) cp-Ti grade 4 RD CBT.

The fracture surface thickness profiles, measured with a micrometer, for cp-Ti in the RD and TD, as well as the cp-Ti RD CBT and AA6016 in the RD, are shown across the width of the post-fracture specimens in Figure 10a–c. The 11.7 mm and 20 mm width specimens showed a typical fracture pattern, with the failure occurring at an oblique angle to the loading direction (Figure 6 for DIC contour plots and pictures of fractured specimens in Appendix C). As the localization and subsequent propagation of the fracture occurred from the center of the specimen, the 11.7 mm and 20 mm cp-Ti specimens, as well as the 11.7 mm AA6016 specimen, exhibited a sharp, “V-shaped” thickness profile, with the lowest thickness value occurring at the center of the specimen. The ≥60 mm width cp-Ti UT specimens, which exhibited a fracture that was perpendicular to the loading direction and included the characteristic cup-and-cone fracture profile, exhibited a similar “V”-shaped thickness profile, though less steep. Note that for all AA6016 specimens, the fracture occurred at an oblique angle to the loading direction (again, Figure 6 and Appendix C), which demonstrated material differences with respect to specimen width.

There was an observable difference between the thickness profiles of cp-Ti CBT (Figure 10d) and UT (Figure 10a,b) specimens. The cp-Ti UT samples ≥60 mm (Figure 10a,b) exhibited notably steeper thickness gradients across the fracture surface compared to CBT samples. The steeper gradients in UT indicate more localized deformation and necking compared to CBT, which is attributed to the fundamental differences in loading conditions. UT induced a stress state that promoted concentrated strain localization, leading to pronounced necking and, consequently, steeper thickness gradients. In contrast, CBT distributed the strain more evenly across the specimen width due to the cyclic, incremental loading. This resulted in less severe thickness variations and provided a delay in the onset of critical necking, providing a good explanation for the higher overall elongation typically observed in CBT compared to UT, as reported in previous studies [36,37,38]. Within cp-Ti UT results, TD specimens (Figure 10b) displayed steeper gradients than RD specimens for the narrower specimen widths (i.e., ≤60 mm). Additionally, the cp-Ti TD exhibited thickness values in the center of the specimen that decreased with increasing width, while RD specimens had the same thickness in the center, where fracture was initiated for all specimen widths (Figure 10a). This anisotropic behavior was consistent with the higher formability, i.e., post-necking deformation, observed in the TD.

Cp-Ti exhibited more significant thickness variations than AA6016 in both UT and CBT, indicating a greater susceptibility to localized deformation for cp-Ti. AA6016 (Figure 10c) demonstrated more uniform thickness profiles across all specimen widths, which showed a more homogeneous deformation process and less sensitivity to localized necking compared to cp-Ti. The higher r-value of cp-Ti promoted greater lateral contraction prior to failure, whereas AA6016, with its lower r-value, experienced less lateral contraction and more pronounced thinning. CBT processing allowed cp-Ti to deform more like AA6016 under UT loading, which provided known and desired material behavior, i.e., lower major strain with increasing specimen width from the more uniform deformation (Figure 8 and Figure 9c).

## 4. Discussion

**Figure 11:**
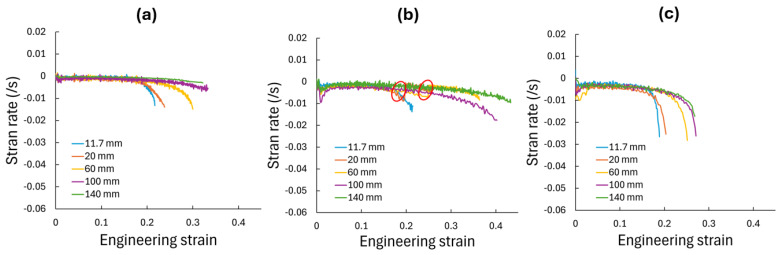
Evolution of center specimen thinning strain rate versus engineering strain for (**a**) cp-Ti grade 4 RD, (**b**) cp-Ti grade 4 TD, and (**c**) AA6016 RD with varying specimen widths from 11.7 mm to 140 mm.

The results presented in this study revealed a significant influence of specimen width on the deformation behavior of cp-Ti grade 4 sheets. Contrary to traditional FLD tenets, wider specimens exhibited higher limit strains at peak stress and increased engineering strain at failure. This trend was observed in both the RD and TD, with TD specimens consistently showing higher formability, i.e., more post-necking deformation. The “apparent” engineering stress–strain curves demonstrated that as specimen width increased from 11.7 mm to 140 mm, the engineering strain at fracture increased substantially. This behavior challenges conventional understanding, where wider specimens exhibit more plane-strain behavior with potentially lower engineering strain at necking, which here is assessed using the peak stress value (Figure 9). The strain distribution analysis provides insights into this unexpected behavior. Narrow specimens (11.7 mm and 20 mm) showed relatively uniform strain distribution along their gauge length. As width increased, a more complex strain pattern emerged, with wider specimens (100 mm and 140 mm) displaying a distinct strain concentration in the center of the specimen. This evolution suggests a transition from a nearly uniaxial stress state in narrow specimens to a more complex, multiaxial stress state in wider specimens. The gradual development of these heterogeneous strain patterns in wider specimens appeared to delay the onset of critical localization that leads to failure, whether that be necking or fracture. This was evident in the thinning strain rates in the center of the specimen (Figure 11). As the specimen width increased, the strain at which localization occurs increased. That is, the rate of thinning in the center of the specimen is moderated by the supporting material on either side, where thinning progresses at a slower rate, which ultimately delays necking and failure compared to narrow specimens that tend to exhibit more homogenous strain patterns across their width, as seen in Figure 6. Furthermore, cp-Ti 20 and 60 mm TD specimens showed increases in the thinning strain rate early in the process (see red circles in Figure 11b) that were shifted back through to a lower strain rate to achieve the expected pattern of higher strains at localization, i.e., increasing strain rate, for wider specimens.

**Figure 12:**
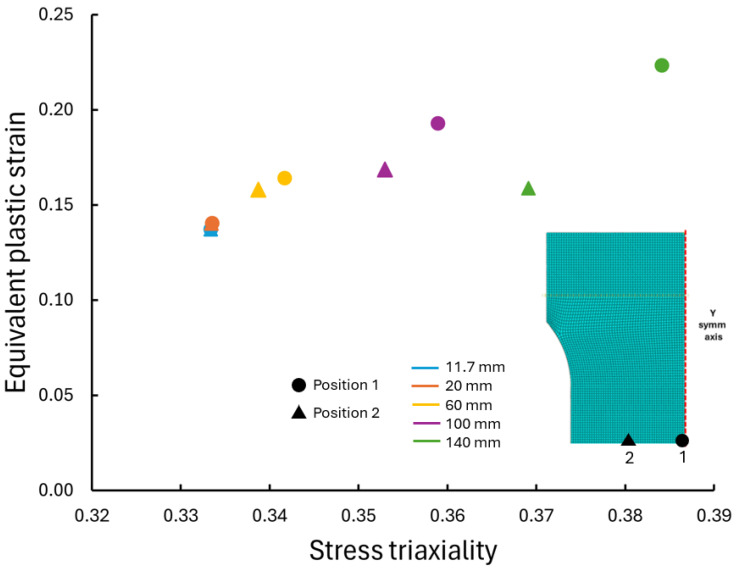
Stress triaxiality with equivalent plastic strain at peak stress (UTS) at two positions across specimen widths for cp-Ti grade 4 RD.

The relationship between stress triaxiality and equivalent plastic strain, extracted from cp-Ti grade 4 RD FE simulations at two distinct positions (center-Position 1, midway-Position 2) across specimen widths, is shown in Figure 12. The stress triaxiality evolved from near uniaxial tension values in narrow specimens (11.7 mm, 20 mm), i.e., ~0.33, toward higher values in wider specimens (100 mm, 140 mm), particularly at the specimen center, although not achieving the theoretical plane strain value of ~0.57. This transition in stress state was accompanied by an increase in equivalent plastic strain with specimen width, providing insight into the observed increase in strain at UTS for wider specimens. The difference in stress triaxiality between the center and midway positions became more pronounced with increasing specimen width, indicating the development of more complex stress states in wider specimens. This heterogeneity in stress state contributes to the higher strains achieved in wider specimens.

These results present an intriguing challenge to traditional FLD and FFL concepts. Conventionally, FLC and FFL curves predict that as the strain state approaches plane strain, the forming limit typically reaches a minimum, i.e., lower major strain. However, these observations revealed a contrasting behavior, i.e., as the specimen width increased and the strain state shifted slightly away from UT and towards plane strain, the major and minor strains at peak stress increased and decreased, respectively (Figure 9). This trend was consistently observed in both the RD and TD, suggesting it is a fundamental characteristic of the material and geometry rather than an isolated anomaly. This same trend was not seen in AA6016 for specimen widths ≤60 mm, with specimen geometry effects causing the 100- and 140 mm specimens not to follow the expected trend. This apparent deviation of cp-Ti from the traditional FLC and FFL behavior has been attributed to several factors, as discussed earlier.

**Figure 13:**
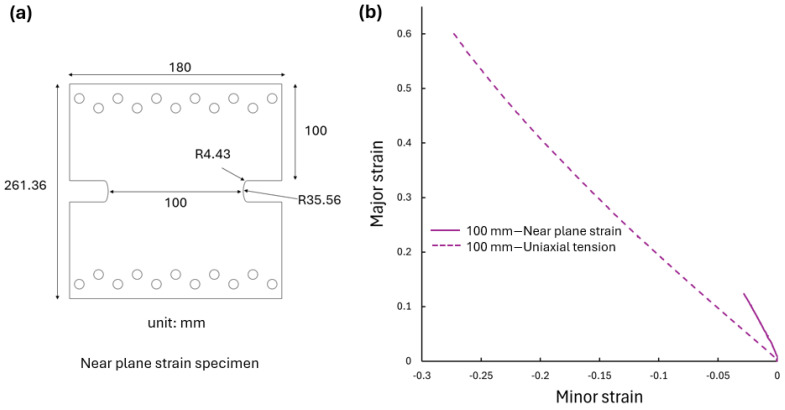
(**a**) Schematic of the near-plane-strain specimen geometry and (**b**) comparison of strain paths between 100 mm wide near-plane-strain and uniaxial tension results for cp-Ti RD.

In addition to the UT tests of specimen geometries in Figure 3, a scaled plane strain test based on a custom design specimen from [39] was conducted using the specimen geometry shown in Figure 13a. This specimen, with a gauge width of 100 mm, featured a much shorter central gauge section designed to induce a near-plane-strain condition. The strain path results for this 100 mm scaled plane-strain specimen were compared with those of the 100 mm UT specimen in Figure 13b. The near-plane strain specimen exhibited a linear strain path much closer to the vertical axis compared to the UT specimen. The maximum major strain achieved in the near-plane-strain condition was lower than that in UT, which is consistent with traditional forming limit concepts.

The current findings on UT complement and extend previous work on CBT of cp-Ti sheets [31]. In both studies, a significant influence of specimen width on deformation behavior was observed. The CBT study revealed decreasing elongation-to-fracture with increasing width, while this current study showed increasing strain at fracture, as well as peak stress, with width for UT testing. This apparent discrepancy can be reconciled by considering the different deformation mechanisms at play. In CBT, the cyclic nature of the loading introduces additional complexities, such as strain reversals and texture evolution [40], which may be more sensitive to width effects. In UT, the absence of cyclic loading allowed for a clearer observation of the intrinsic material response to width-dependent stress states. The combined insights from both studies suggest that the formability of cp-Ti is highly dependent on both the loading path and the specimen geometry.

**Figure 14:**
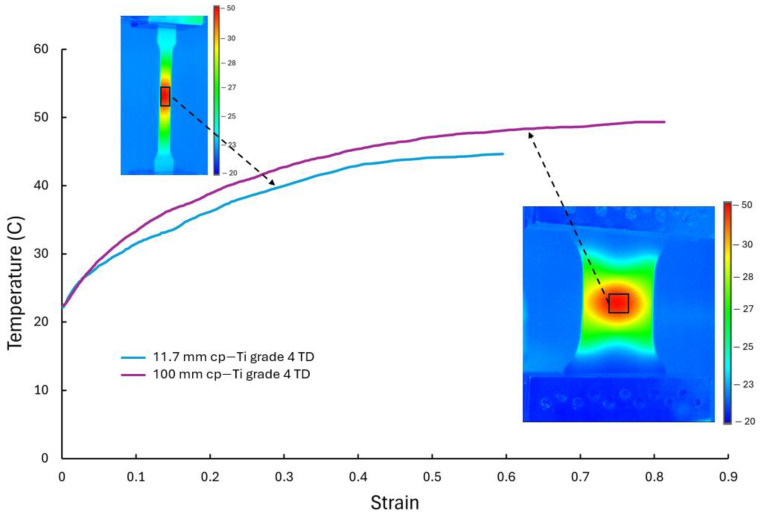
Temperature evolution versus engineering strain for the cp-Ti grade 4 TD specimens of 11.7 and 100 mm widths, with inset thermal images showing heat distribution patterns at the final frame prior to fracture.

Finally, the effect of temperature as a possible factor in the behavior of wider cp-Ti sheets was investigated. Recent studies have shown the importance of considering temperature evolution during deformation, with Renault et al. [41] demonstrating through thermo-mechanical simulations how local temperature changes can significantly affect deformation behavior, albeit in different material systems. The temperature evolution during testing was monitored for the cp-Ti grade 4 TD specimens for both narrow (11.7 mm) and wide (100 mm) samples (Figure 14). Both specimen widths showed a temperature increase during deformation, with the 140 mm specimen showing a higher temperature due to the higher thermal mass of the larger specimen geometry. For the 11.7 mm specimen, the temperature rose to about 44 °C and plateaued at higher strains. The 100 mm specimen showed a continuous temperature increase up to approximately 49 °C. The thermal images revealed localized heating patterns, where the narrow specimen showed a concentrated heat zone along its width and length, while the wide specimen displayed a more diffuse, central heat concentration, which is consistent with the higher strain in this region. However, the maximum temperature rise (approximately 25 °C above ambient) is not expected to significantly alter the mechanical properties of cp-Ti grade 4 during the test duration. Therefore, these temperature effects are not considered to substantially impact the main findings of the study. Nevertheless, thermally activated dislocation motion can be aided by slight increases in temperature [42].

## 5. Conclusions and Future Work

Commercially pure Titanium (cp-Ti) grade 4 is a material of interest in, e.g., aerospace, chemical processing, and biomedical applications. Therefore, accurate material characterization is required. This research revealed unexpected behavior in cp-Ti samples as specimen width increased. Typically, moving from uniaxial tension towards plane-strain conditions (by increasing specimen width) should result in decreased major strain at localization (assessed at peak stress values) and fracture. However, cp-Ti UT samples did not follow this expected trend. The deviation from expected behavior appeared to be due to the development of heterogeneous strain fields in wider specimens. In contrast, by using CBT processing, cp-Ti grade 4 exhibited behavior more consistent with traditional forming limit concepts, both FLD and FFL, i.e., decreased major strain and increased (less negative) minor strain, with increasing specimen width. This contrast between UT and CBT results highlights the significant impact of loading conditions on material behavior, which was also demonstrated by conducting experiments on AA6016-T4 as well, which demonstrated expected FLC behavior for specimen widths ≤ 60 mm. The behavior of the AA6016 is explained by its low r-value and tendency to exhibit greater thinning and less lateral contraction for wider specimen geometries, leading to early failure for more plane-strain conditions. In contrast, the high r-value cp-Ti material resisted the plane-strain condition, even for the widest specimen, and instead exhibited a gradient of thinning strain across its width, with greater magnitudes in the center of the specimen and much lower ones on either side. The gradient of strain delayed localization in the specimen center, as previously discussed, and resulted in the surprising outcome of greater elongation in simple tension than was observed in narrower specimens. Thus, the CBT test’s ability to create more uniform deformation across even the wide CP-Ti specimens, as evidenced by consistent thinning across the specimen width for cp-Ti, produces more predictable formability, with respect to conventional FLD and FFL results where conditions closer to plane-strain exhibit lower formability.

These findings open several avenues for future research and have important practical implications. While this current study focuses on deformation behavior and formability comparisons between UT and CBT, future investigations could benefit from analyzing various fracture criteria, such as Cockcroft–Latham and Rice–Tracy, to better understand the failure mechanisms. These criteria could provide additional insights into how stress triaxiality and loading path influence fracture behavior across different specimen widths. Such analysis might help explain the observed differences in failure modes between narrow and wide specimens and potentially offer new perspectives on the relationship between specimen geometry and fracture characteristics in cp-Ti grade 4 sheets. In addition, further studies on the evolution of microstructure and texture during the deformation of specimens with different widths could provide deeper insights into the underlying mechanisms responsible for the observed size effects. From a practical standpoint, the enhanced formability observed in wider specimens suggests potential benefits in designing forming processes that leverage these geometry effects.

## Figures and Tables

**Table 1:** Chemical composition of 1 mm thick cp-Ti grade 4 sheets (wt.%) [31].

C	Fe	H	N	O	Ti
0.005	0.19	0.0006	0.004	0.25	Balance

**Table 2:** Mechanical properties of cp-Ti grade 4 sheet in different testing directions.

Direction	Young’s Modulus (GPa)	Yield Strength (MPa)	Ultimate Tensile Strength (MPa)	Engineering Strain at UTS	Engineering Strain at Failure
RD	109	574	696	0.1395	0.218
TD	118	670	725	0.1208	0.217

**Table 3:** Detailed dimensions for each specimen, in mm, for dimension labels in Figure 3.

Specimen	Width (W)	Length (L)	Gauge Width (B)	Radius (R)	Gauge Length (G)
11.7	20	183.34	11.7	12.7	75
20	60	274.44	20	60	
60	100		60		
100	140		100		
140	180		140		

**Table 4:** Strain ratio values for all specimen widths.

Specimen Width (mm)	cp-Ti Grade 4 RD	cp-Ti Grade 4 TD	AA6016-T4
11.7	1.31	2.47	0.83
20	1.26	2.55	0.73
60	1.41	2.42	0.64
100	1.31	2.04	0.57
140	0.94	1.52	0.49

**Figure A1:**
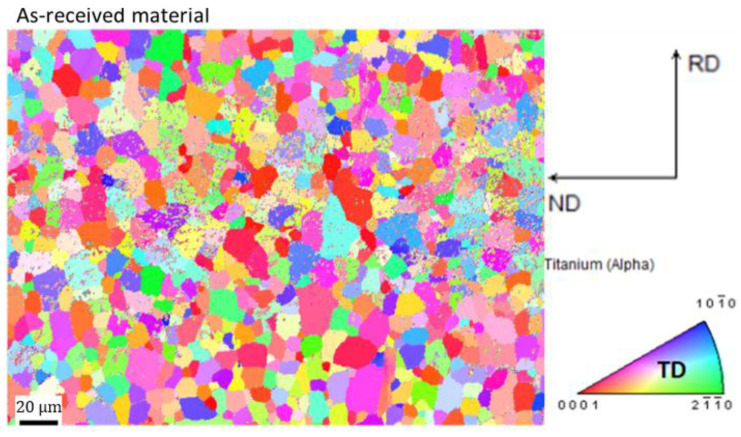
Inverse pole figure (IPF) map of undeformed grain structure in cp-Ti grade 4 sheets [32,33].

**Figure A2:**
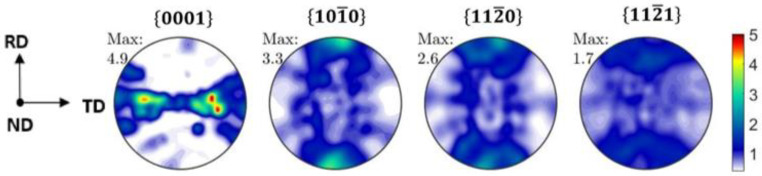
Stereographic pole figures showing texture in undeformed cp-Ti grade 4 sheets [32,33].

**Figure A3:**
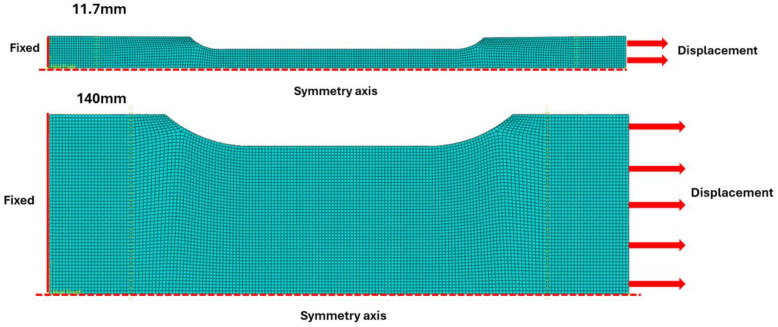
FE meshes for one half of the 11.7 mm and 140 mm specimens consisting of C3D8R elements along with the boundary conditions constructed in Abaqus for simulating the uniaxial tensile tests.

**Figure A4:**
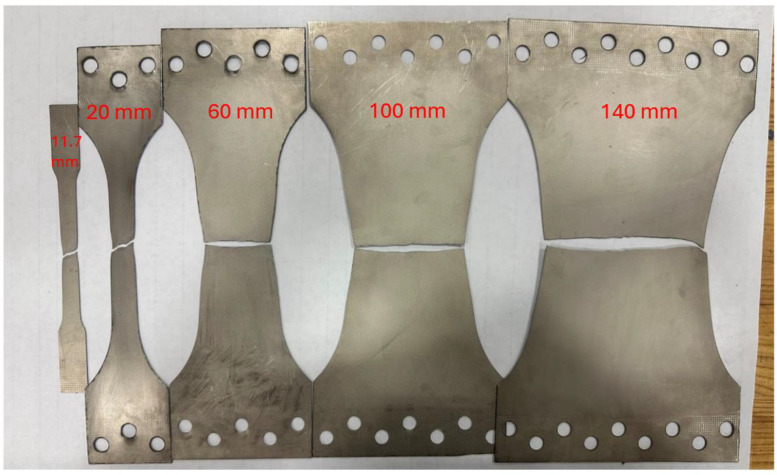
Photograph of cp-Ti grade 4 RD fractured specimens for all widths.

**Figure A5:**
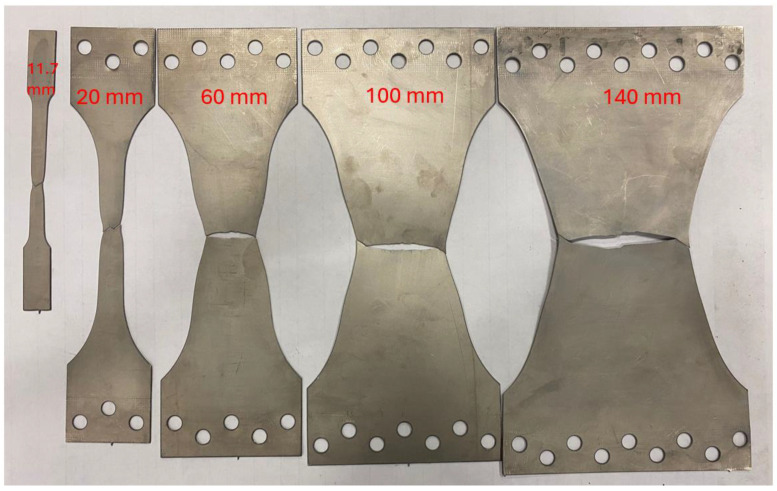
Photograph of cp-Ti grade 4 TD fractured specimens for all widths.

**Figure A6:**
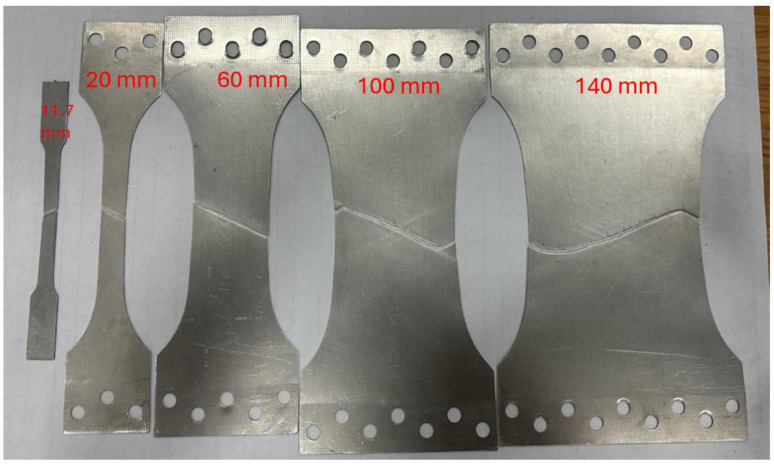
Photograph of AA6016-T4 RD fractured specimens for all widths.

## Data Availability

The raw data supporting the conclusions of this article will be made available by the authors on request.

## Appendix A. Initial Microstructure and Texture Analysis of cp-Ti Grade 4 Sheet

Figure A1 illustrates the inverse pole figure (IPF) map showing the undeformed grain structure in the sheets of cp-Ti grade 4. The colors in the IPF triangle represent the orientation of the TD sheet axis relative to the frame of individual grains per map [32,33]. This revealed the equiaxed grain structure of the as-received material. Figure A2 illustrates the stereographic pole figures showing texture in the undeformed sheets of cp-Ti (grade 4) [32,33]. The pole figures indicate a typical rolled and annealed texture for cp-Ti, with basal poles tilted away from the normal direction toward the TD, which helps explain the observed anisotropic behavior and higher formability in the TD noted in our study.

## Appendix B. Finite Element Model Setup

Figure A3 illustrates the finite element model setup used for simulating the UT tests with varying widths. The model leverages half symmetry, with appropriate symmetry boundary conditions applied along the longitudinal centerline, to reduce computational cost while maintaining accuracy.

## Appendix C. Fractured Specimen

Figure A4, Figure A5 and Figure A6 present the post-test fractured specimens for cp-Ti RD, cp-Ti TD, and AA6016 RD, respectively. These images provide valuable insights into the deformation and fracture behavior of the materials across different specimen widths.
